# Live Birth After Oocyte Donation In Vitro Fertilization Cycles in Women With Endometriosis: A Systematic Review and Meta-Analysis

**DOI:** 10.1001/jamanetworkopen.2023.54249

**Published:** 2024-01-31

**Authors:** Alessio Paffoni, Maíra Casalechi, Dominique De Ziegler, Ettore Cicinelli, Edgardo Somigliana, Paola Viganò, Amerigo Vitagliano

**Affiliations:** 1 Infertility Unit, ASST Lariana, Cantù (Como), Italy; 2 Human Reproduction Unit of the Hospital das Clínicas, Universidade Federal de Minas Gerais, Belo Horizonte, Brazil; 3 Department of Obstetrics, Gynecology and Human Reproduction, Hospital Foch, Suresnes, France; 4 First Unit of Obstetrics and Gynecology, Department of Interdisciplinary Medicine, University of Bari, Bari, Italy; 5 Infertility Unit, Department of Obstetrics and Gynecology, Fondazione IRCCS Ca’ Granda Ospedale Maggiore Policlinico, Milano, Italy

## Abstract

**Importance:**

Although multiple mechanisms have been proposed to explain the infertility related to endometriosis, there are no conclusive data on the association of endometriosis with endometrial receptivity. The oocyte donation model in assisted reproduction technology (ART) cycles can clarify this issue.

**Objective:**

To explore the association of a history of endometriosis with ART outcomes in recipients of oocyte donation.

**Data Sources:**

In this systematic review and meta-analysis, electronic databases were searched from inception until August 31, 2023, using combinations of relevant keywords. Moreover, we retrieved data from the databases of the Society for Assisted Reproductive Technology (SART) in the US and the Human Fertilization and Embryology Authority (HFEA) in the United Kingdom.

**Study Selection:**

Observational studies were included if they investigated the impact of endometriosis on ART outcomes with donor oocytes.

**Data Extraction and Synthesis:**

Publicly available data related to ART from various sources were gathered, and a retrospective aggregate and nonaggregate analysis using registries of in vitro fertilization cycles with oocyte or embryo donation was conducted.

**Main Outcomes and Measures:**

The primary outcome was live birth rate (LBR) following oocyte donor cycles. The effect measures of comparisons between groups are presented as odds ratios (ORs) with a 95% CI.

**Results:**

This study analyzed 7212 oocyte donation cycles from 4 studies for the meta-analysis, along with 162 082 cycles from 2 registries (137 182 from SART and 24 900 from HFEA). No significant differences between the groups were observed in the meta-analysis of published data after adjusting for confounding factors (OR, 0.54; 95% CI, 0.19-1.57). A statistically significant lower LBR was identified in women with endometriosis when analyzing the aggregate data from SART and HFEA databases (OR, 0.89; 95% CI, 0.81-0.97).

**Conclusions and Relevance:**

This study found a modest decrease in LBR among women with a history of endometriosis, although only results from the pooled analysis of registry data and not those from the meta-analysis reached statistical significance. These findings suggest that a marginal impairment of uterine receptivity may contribute to infertility mechanisms in women affected by endometriosis.

## Key Points

**Question:**

Is a history of endometriosis associated with lower success rates in recipients of an in vitro fertilization oocyte donation program?

**Findings:**

This systematic review and meta-analysis includes data from 2 diverse sources (7212 oocyte donation cycles from published studies and 162 082 cycles from 2 registries). Both analyses revealed a modest reduction in live birth rates among recipients with endometriosis, although only results from the pooled analysis of registry data reached statistical significance.

**Meaning:**

These findings suggest that uterine receptivity to in vitro fertilization may be marginally affected in women with a history of endometriosis.

## Teaser

This systematic review and meta-analysis explores the association of a history of endometriosis with assisted reproduction technology outcomes in recipients of in vitro fertilization (IVF) oocyte donation.

## Introduction

Endometriosis affects 10% to 15% of women of reproductive age, and up to 25% to 50% of women with infertility have endometriosis.^1,2,3,4^ Currently, in women affected, infertility can be managed with either surgery or assisted reproduction technology (ART).^5^ In women with endometriosis who undergo autologous in vitro fertilization (IVF) procedures, success rates have been reported to be lower compared with women without the disease.^3^ An increased risk of unexpected poor response to ovarian stimulation and a lower number of retrieved oocytes beyond the well-known detrimental effects of surgery for ovarian endometriomas have been associated with the disease.^6^ Therefore, women with endometriosis face an elevated likelihood of being directed toward donor egg ART cycles. It remains uncertain, however, whether this approach can fully eliminate the adverse impacts of the condition on endometrial receptivity.^7^

The concept that the uterine environment of women with endometriosis may affect the embryo implantation process remains controversial.^8^ Endometrial changes, primarily or secondarily associated with inflammation related to the disease, are believed to contribute, at least in part, to impaired receptivity.^9^ This concern may be particularly crucial in donor egg cycles, possibly resulting in lower live birth rate (LBR) in recipients with endometriosis compared with those with other indications. Therefore, the primary objective of this study was to assess whether endometriosis may impact ART success rates in recipients of oocyte donation cycles through a systematic review and meta-analysis of published data. In addition, we conducted an original analysis of the data from the Society for Assisted Reproductive Technology Clinic Outcome Reporting System (SART CORS) and Human Fertilisation and Embryology Authority (HFEA) clinical databases.

## Methods

### Systematic Review

#### Study Protocol

This systematic review with meta-analysis of published data was prospectively registered in PROSPERO (CRD42023399558). The recommendations of the Preferred Reporting Items for Systematic Reviews and Meta-analyses (PRISMA) reporting guidelines for systematic reviews and meta-analyses were followed.^10^ Since we used publicly available registry data, ethical approval and informed consent were not needed, in accordance with 45 CFR §46. The SART and HFEA registries prioritize patient privacy by deidentifying data before public access. Information sources, search, and eligibility criteria; study selection and data collection process; assessment of the risk of bias; and grading of evidence are reported in the eAppendix in Supplement 1. Modified Newcastle-Ottawa scoring items are shown in eTable 1 of Supplement 1.

#### Data Synthesis

The LBR following IVF embryo transfers with donor oocytes in recipient women with and without endometriosis was our primary outcome. Secondary outcomes included the evaluation of clinical pregnancy rate (CPR), implantation rate (IR), and miscarriage rate (MR) in the same women. Measures included LBR per embryo transfer, defined as the delivery of 1 or more living infants; CPR per embryo transfer, defined as number of pregnancies diagnosed by ultrasonographic visualization of 1 or more gestational sacs or definitive clinical signs of pregnancy; IR per transferred embryos, defined as the number of gestational sacs on transvaginal ultrasonography divided by the number of embryos transferred; and MR per clinical pregnancy, defined as fetal loss prior to the 20th week of gestation divided by the number of clinical pregnancies.^11,12^

### Statistical Analysis

Two authors (A.V. and A.P.) independently conducted statistical analysis using Review Manager software version 5.4 (Nordic Cochrane Centre, Cochrane Collaboration). Discrepancies in results were resolved through consensus. We used the random effects model (DerSimonian and Laird method) for all analyses. Odds ratios (ORs) with a 95% CI represented effect measures. In the unadjusted model, we combined dichotomous data to calculate an unadjusted OR. Absolute risk difference (RD) with a 95% CI was used to express differences in observed risks between groups. Adjusted ORs were used when available, and we calculated a pooled adjusted OR using the generic inverse variance method. Pooled ORs were derived from the natural logarithm (LN) of individual studies’ OR and corresponding 95% CI. The SE for LN (OR) was calculated from the 95% CI using the following formula: SE = [LN (upper CI limit) − LN (lower CI limit)] / 3.92. Adjusted confounders for the included studies^13,14,15,16^ are reported in Table 1. Heterogeneity was assessed using the Higgins *I*^2^ statistic, and results were presented graphically with forest plots. We planned sensitivity analyses to test result robustness and investigate sources of statistical heterogeneity by excluding studies with higher bias risk if at least 3 studies were included. However, owing to the limited number of studies, we could not perform these analyses. We conducted a subgroup analysis to differentiate between adjusted and unadjusted models and evaluate differences in patient subgroups undergoing fresh or frozen-thawed oocyte donation cycles. Publication bias was assessed following Cochrane handbook recommendations.^17^

**Table 1.** General Characteristics of the Studies Included

Source	Study design	Country	Total No. of patients	Main inclusion criteria	Embryo transfer cycle	Age, mean (SD), y	Confounders adjusted
Sung et al,^15^ 1997	Retrospective study (medical records)	US	239	Oocyte recipients with (n = 55) and without (n = 184) endometriosis	Fresh embryo transfer; day 2 embryos; HRT cycles; No. of embryos transferred not determined but it was not only single embryo transfer	Endometriosis, 41.8 (4.3) y; controls, 41.5 (5.6) y	No adjustment
Díaz et al,^14^ 2000	Prospective matched controlled study	Spain	58	Oocytes recipients with (n = 25) and without (n = 33) endometriosis	Fresh embryo transfer; day 2 embryos; HRT cycles; No. of embryos transferred not determined but it was not only single embryo transfer	Endometriosis, 35.0 (3.4) y; controls, 38.5 (4.9) y (*P* < .05)	No adjustment
Prapas et al,^13^ 2012	Prospective, matched controlled study	Greece	240	Menopausal oocytes recipients with (n = 120) and without (n = 120) endometriosis	Fresh embryo transfer; day 3 embryos; up to 3 embryos per transfer; HRT cycle	Controls, 45.4 (2.7) y; endometriosis, 37.3 (4.4) y (*P* < .05)	Implantation, clinical pregnancy, miscarriage, ongoing pregnancy, and live birth rates were adjusted for age and BMI
Kamath et al,^16^ 2022	Retrospective study (Registry analysis)	United Kingdom	6675	Oocytes recipients with (n = 758) and without (n = 5917) endometriosis	Fresh and frozen cycles; before day 5 and day 5 or later embryos; not only single embryo transfer; endometrial preparation not determined	NA	Live birth rate was adjusted for number of previous IVF cycles, previous live birth, IVF or ICSI, period of treatment, day of embryo transfer, number of embryos transferred and fresh or frozen embryo transfer cycles

Abbreviations: BMI, body mass index; HRT, hormone replacement therapy; ICSI, intracytoplasmic sperm injection; IVF, in vitro fertilization; NA, not applicable.

For the analysis of data from clinical registries, publicly available data related to ART from various sources were gathered, and a retrospective analysis using registries of IVF cycles with oocyte or embryo donation was conducted.^13,14,15,16,18,19,20,21,22^ We obtained data from the US SART and UK HFEA databases, which matched our study’s focus. These databases contain extensive IVF information from various clinics on oocyte and/or embryo recipients with and without endometriosis, considering both fresh and frozen cycles. Consistently with the aim of the study, we focused on transfer cycles.

The SART registry data from 2014 to 2020 covered various donor egg cycles, providing key variables like diagnosis of endometriosis, transfer counts, average embryos transferred, single embryo transfer percentage, IR, and LBR per individual year. These variables were essential for calculating comprehensive implantation and LBRs.

For the HFEA registry (2010-2018), we calculated LBRs and IRs for endometriosis cases and nonendometriosis controls. The HFEA data set included main cycle characteristics, and we excluded cycles with no viable embryos, no embryo transfer, cycles with preimplantation genetic testing, and cycles or entries with missing information regarding donor age. Double donation (sperm and oocyte) cycles and embryo donation cycles were considered as a single group. Data sets were analyzed using binary logistic regression to compare women with and without endometriosis. Categorical variables were assessed using a χ^2^ test. Confidence intervals for proportions were computed using a binomial distribution. Two-sided *P* < .05 was considered statistically significant. After individually analyzing each registry, the acquired data were subsequently aggregated in a meta-analysis using the same method detailed above for the pooled analysis of published literature data.

## Results

### Study Selection for Meta-Analysis

To explore the association of endometriosis with ART outcomes in recipients of oocyte donation, we used 2 research strategies: a meta-analysis of available observational studies and a retrospective analysis of nonaggregate and aggregate data from SART and HFEA registries of IVF donor cycles (162 082 total cycles; 24 900 from HFEA and 137 182 from SART). For the first strategy, following the initial screening of titles and abstracts, 9 records were evaluated in detail for eligibility. Of these, 5 studies were excluded for the following reasons: 1 study^23^ was a review article; another study^24^ was deemed inappropriate for inclusion because of its focus on comparing characteristics between pregnant and nonpregnant women following oocyte donation, rather than on the main topic of interest; and 3 studies^18,25,26^ were excluded because the authors, despite being contacted via email, did not provide the necessary data for our analysis. Consequently, 4 studies^13,14,15,16^ met the inclusion criteria and were incorporated into the meta-analysis (eFigure 1 in Supplement 1).

### Included Studies

The retained studies included a total number of 7212 donor transfer cycles, including 958 women with endometriosis and 6254 controls (Table 1). Two studies were prospective,^13,14^ and 2 were retrospective.^15,16^ In the studies by Díaz et al^14^ and Prapas et al,^13^ the oocyte recipients in the endometriosis group and the controls were matched for the same donor (ie, each donor donated to a woman with endometriosis and a woman without endometriosis). The study by Kamath et al^16^ was based on the HFEA UK clinical registry. The study by Sung et al^15^ was a retrospective analysis of data obtained from medical records. Characteristics of included studies are summarized in Table 1.

### Patients

In the study by Kamath et al,^16^ no information was provided about the diagnostic technique or the stage of the disease. The patients with endometriosis had stage III and IV endometriosis according to the American Society for Reproductive Medicine classification in the study by Díaz et al^14^ (25 patients), and variable stages in the studies by Prapas et al^13^ (2 cases with stage I, 28 cases with stage II, 49 cases with stage III, and 41 cases with stage IV endometriosis) and Sung et al^15^ (18 cases with stage I or II, and 37 cases with stage III or IV). The diagnosis of endometriosis was confirmed laparoscopically in 3 studies.^13,14,15^ However, in the study by Kamath et al,^16^ no information was provided regarding the diagnostic method for endometriosis. The control group consisted of women without endometriosis in all the studies. In the study by Prapas et al,^13^ women were premenopausal and younger than 50 years. In the study by Díaz et al,^14^ the patients had a history of failed IVF, low response to ovarian stimulation, and ovarian failure. In the study by Sung et al,^15^ the patients underwent their first oocyte recipient attempt without further specified characteristics. In the study by Kamath et al,^16^ controls were patients undergoing oocyte donation without a specific female indication, with male factor infertility.

### Oocyte Donation Cycles

Among the 4 studies, 3 included only fresh oocyte donation cycles, whereas in the study by Kamath et al,^16^ 69% of the cycles were fresh transfers and the remaining were frozen embryos cycles. The technique for endometrial preparation was described in all studies,^13,14,15^ except for Kamath et al.^16^ In 2 studies, endometrial preparation protocol involved oral estradiol valerate with incremental doses (up to 6 mg per day), followed by vaginal progesterone (800 mg per day in Díaz et al^14^ and 600 mg per day in Prapas et al^13^) starting from the day of oocyte retrieval from the donor. In the study by Sung et al,^15^ an incremental dose of transdermal estradiol (0.2-0.4 mg per day) was used, followed by intramuscular progesterone (50-150 mg per day) starting from the day of the donor’s oocyte pick-up. In the studies by Díaz et al^14^ and Prapas et al,^13^ at least 8 oocytes were donated, whereas this information was not provided by Kamath et al^16^ and Sung et al.^15^ In 3 studies, embryos were transferred at cleavage stage (48 hours after oocyte retrieval in Díaz et al^14^ and Sung et al,^15^ and 72 hours after oocyte retrieval in Prapas et al^13^) or both at the cleavage and blastocyst stages.^16^ The average number of embryos transferred was 1.6 in the study by Kamath et al,^16^ 2.1 in Prapas et al,^13^ 3.3 in Sung et al,^15^ and 4 in Díaz et al.^14^

### Assessment of the Risk of Study Bias

#### Sample Representativeness

Two studies^13,16^ were judged to be at low risk of bias for sample representativeness. Other studies were judged to be at high risk of bias (eTable 2 in Supplement 1).

#### Sampling Technique

Two studies^15,16^ had adequate sampling strategy (consecutive). Other studies did not provide data.

#### Ascertainment of Endometriosis Diagnosis

One study^16^ did not report information (high risk of bias). Other studies were at low risk of bias^13,15^ (eTable 2 in Supplement 1).

#### Quality of Description of the Population

One study was considered at high risk of bias because it did not provide information on the indications for oocyte donation in the control group.^15^ Another study did not report information on the type of endometrial preparation performed in the recipients.^16^ In addition, both studies did not provide information on the average number of oocytes donated per cycle.^15,16^

#### Incomplete Outcome Data

One study provided incomplete outcome data^15^ (eTable 2 in Supplement 1). Taking into account all the domains evaluated, only 1 study was considered at high risk of bias^15^ (eTable 2 in Supplement 1).

### Meta-Analysis

#### Primary Outcome

**Figure 1.**
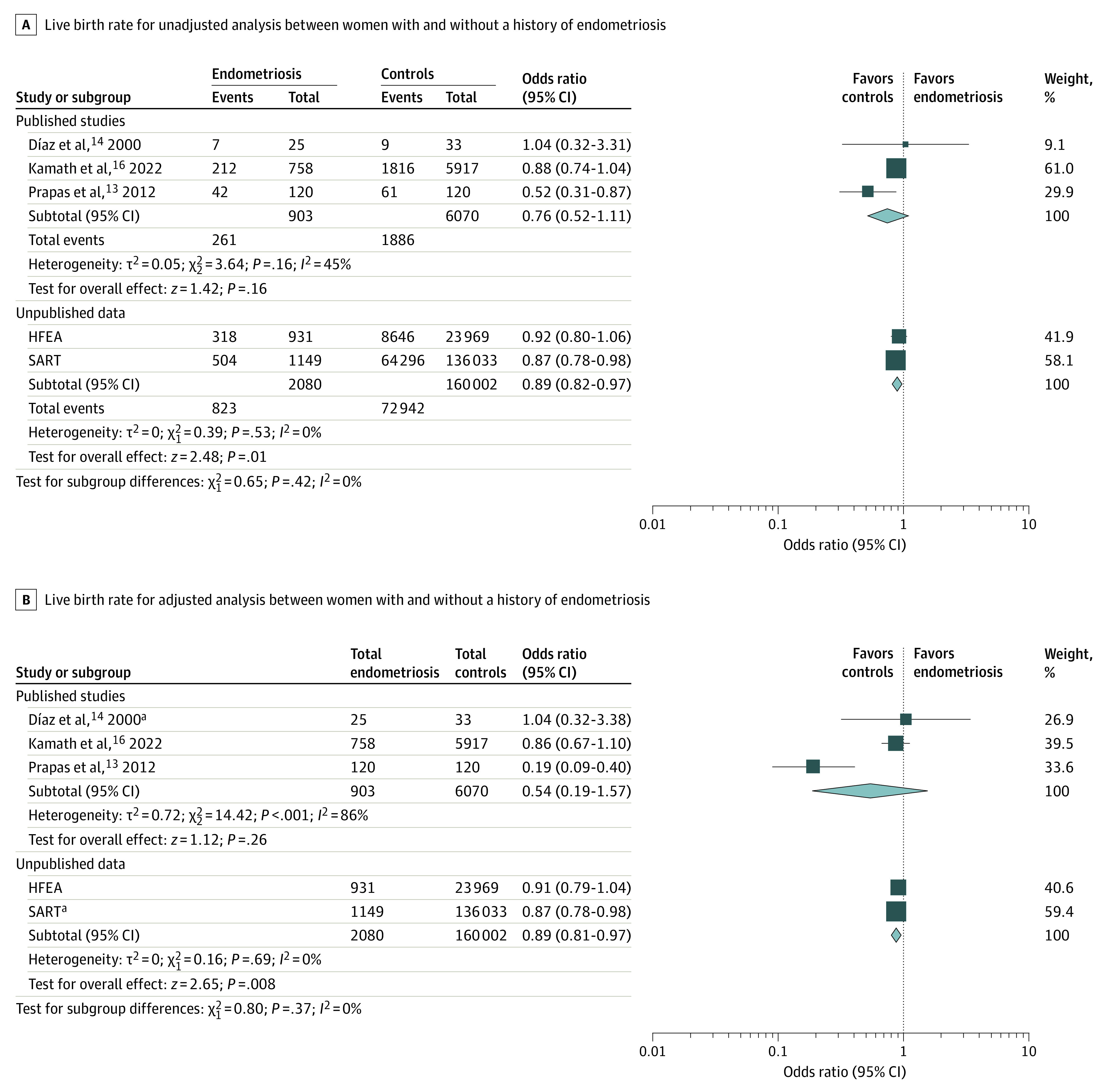
Forest Plots for Live Birth Rate for Unadjusted and Adjusted Analysis Between Women With and Without a History of Endometriosis as Recipients of Egg Donation Assisted Reproductive Technology Cycles Data from published studies and those from registries (Society for Assisted Reproductive Technology [SART] and Human Fertilization and Embryology Authority [HFEA]) were pooled separately. Squares in panel A refer to dichotomous variables. Squares in panel B indicate the inclusion of log odds ratio and the SE of the log odds ratio in the meta-analysis. Size of the squares depends on the weight assigned to each study in the analysis. Diamonds denote the overall effect estimates; the width of the diamond indicates the 95% CI around the combined effect. ^a^Refers to unadjusted data or source in which adjustment was not possible.

Although data for the quantitative analysis were derived from 4 studies, the variability in the outcomes reported by each study did not allow us to aggregate data from all the studies. The aggregate analysis for the primary outcome was based on 6973 patients from 3 studies (903 women with endometriosis and 6070 controls). The unadjusted model showed no significant reduction in LBR per embryo transfer among women with endometriosis (OR, 0.76; 95% CI, 0.52 to 1.11), with an RD of −0.06 (95% CI, −0.15 to 0.03) (Figure 1A). Similarly, incorporating adjusted data into the analysis, the differences between the groups were not significant (OR, 0.54; 95% CI, 0.19 to 1.57) (Figure 1B). The subgroup analysis based on the type of oocyte donation cycles (fresh vs frozen) did not show significant differences between the groups in terms of LBR per embryo transfer, both in the unadjusted and mixed models (eFigure 2 in Supplement 1).

#### Secondary Outcomes

**Figure 2.**
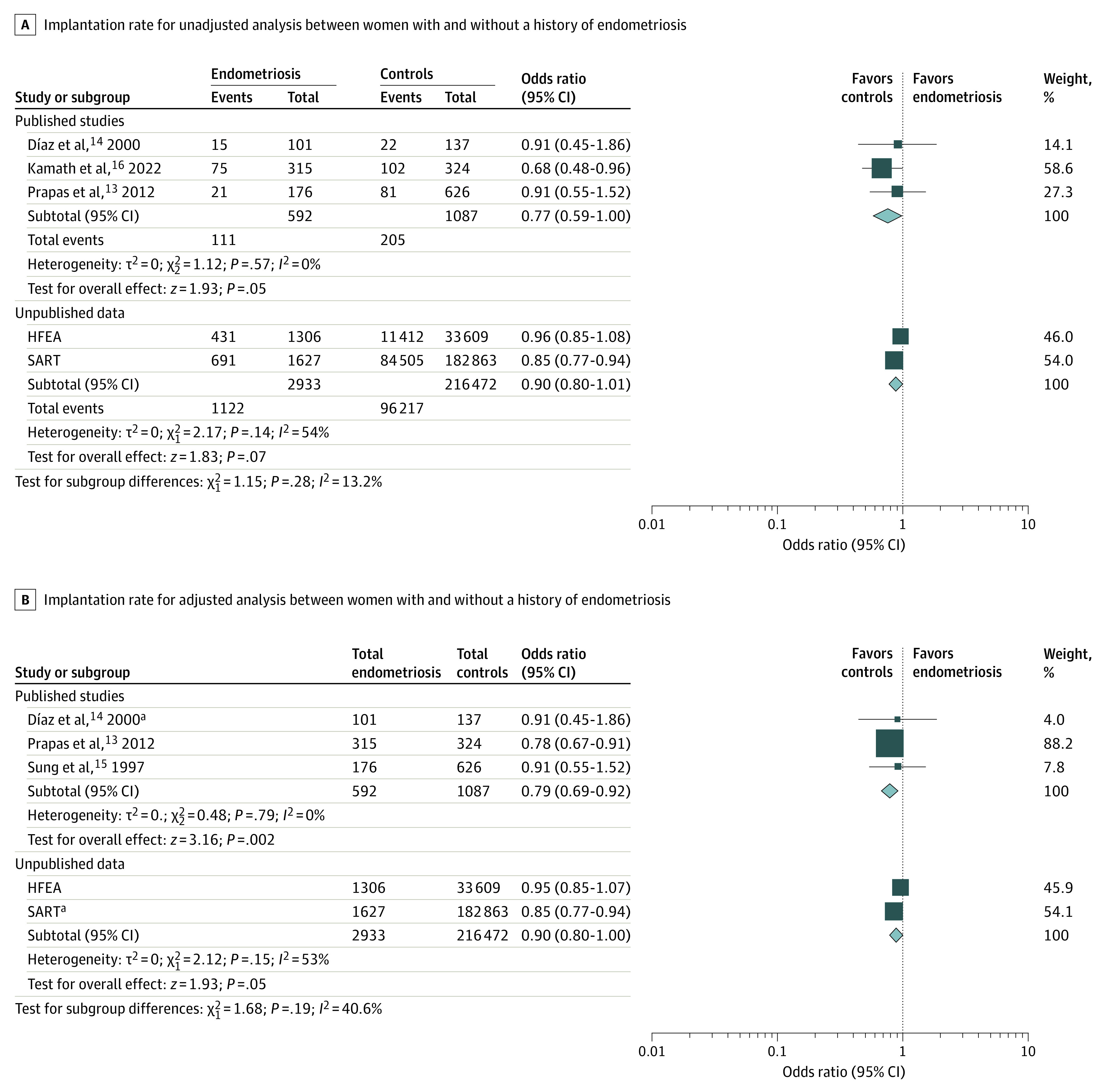
Forest Plots for Implantation Rate for Unadjusted and Adjusted Analysis Between Women With and Without a History of Endometriosis as Recipients of Egg Donation Assisted Reproductive Technology Cycles Data from published studies and those from registries (Society for Assisted Reproductive Technology [SART] and Human Fertilization and Embryology Authority [HFEA]) were pooled separately. Squares in panel A refer to dichotomous variables. Squares in panel B indicate the inclusion of log odds ratio and the SE of the log odds ratio in the meta-analysis. Size of the squares depends on the weight assigned to each study in the analysis. Diamonds denote the overall effect estimates; the width of the diamond indicates the 95% CI around the combined effect. ^a^Refers to unadjusted data or source in which adjustment was not possible.

The unadjusted model of the data from 3 studies involving 537 patients found no significant reduction in IR (OR, 0.77; 95% CI, 0.59 to 1.00; RD, −0.03; 95% CI, −0.08 to 0.01) (Figure 2A) or CPR (OR, 0.70; 95% CI, 0.48 to 1.03; RD, −0.08; 95% CI, −0.16 to 0.01). No differences were observed in terms of MR (OR, 1.31; 95% CI, 0.55 to 3.13). The inclusion of adjusted data in the aggregate analysis showed a significant reduction in IR in women with endometriosis (OR, 0.79; 95% CI, 0.69 to 0.92) (Figure 2B), with no effects on CPR (OR, 0.61; 95% CI, 0.24 to 1.53) and MR (OR, 1.16; 95% CI, 0.42 to 3.22).

#### Quality of Evidence

The quality of evidence was rated as low for LBR, very low for CPR, IR, MR. See details in eTable 3 in Supplement 1.

### Data Retrieved From Registries

#### SART Registry

On the basis of the completeness of the data, the SART registry was consulted for the period 2014 to 2020. In this period, there were 1149 transfers involving donor eggs for women with a history of endometriosis, which included 201 transfers using donated embryos. For women without endometriosis, there were 136 033 transfers with donated oocytes and 11 589 transfers using donated embryos in the same period. In the endometriosis group, a mean of 1.4 embryos per transfer were used in the donor egg procedures and 1.6 embryos per transfer in the donated embryo procedures. Conversely, in the group without endometriosis, a mean of 1.6 embryos per transfer were used in the oocyte donation procedures and 1.8 embryos per transfer in the donated embryo procedures. When evaluating the outcomes according to these transfers, patients with endometriosis showed significantly lower LBR (absolute difference, −3.4%; OR, 0.87; 95% CI, 0.78-0.98) (Figure 1) and IR (absolute difference, −3.7%; OR, 0.85; 95% CI, 0.77-0.94) (Figure 2) compared with unaffected women.

#### HFEA Registry

The HFEA registry was consulted for the period 2010 to 2018 because the data were found to be aggregated in this period, and information was not available thereafter. According to the exclusion criteria, cycles with the following characteristics were removed from the data set based on the HFEA registry: absence of embryos to be transferred (3252 cycles), unknown egg donor age (2256 cycles), and preimplantation genetic testing cycles (185 cycles). A total of 24 900 embryo transfer cycles were retained in the data set including 931 endometriosis cases and 23 969 individuals without a diagnosis of endometriosis (eTable 4 in Supplement 1).

When examining data set entries across various age categories, the LBRs and IRs showed no significant differences between women with and without endometriosis (eTable 5 in Supplement 1). Overall, the IR was 33.0% in patients with endometriosis and 34.0% in those without endometriosis (absolute difference, −1.0%), whereas the LBR per embryo transfer was 34.2% in patients with endometriosis and 36.1% in patients without endometriosis (absolute difference, −1.9%). Results according to the type of procedure in endometriosis patients and unaffected women are reported in Table 2.

**Table 2.** Implantation Rate and Live Birth Rate in Oocyte Donation Cycles Reported in the SART and HFEA Registries

Registry and type of oocytes	Implantation rate, % (95% CI)		*P* value	Live birth rate per embryo transfer, % (95% CI)		*P* value
	Endometriosis	Nonendometriosis		Endometriosis	Nonendometriosis	
SART						
Fresh oocytes	51.4 (45.8-56.9)	52.8 (52.2-53.3)	.62	56.0 (49.0-62.9)	55.8 (55.2-56.5)	.95
Frozen oocytes	35.8 (29.6-42.3)	43.9 (43.2-44.5)	.01	40.0 (32.2-48.2)	45.6 (44.9-46.3)	.16
Thawed embryos	41.2 (38.2-44.2)	45.1 (44.5-45.4)	.01	41.5 (38.0-44.9)	45.5 (45.2-45.8)	.02
Total	42.5 (40.1-44.9)	46.4 (46.2-46.6)	.002	43.9 (41.0-46.8)	47.3 (47.0-47.5)	.02
HFEA						
Fresh oocytes	35.2 (31.9-38.6)	36.6 (35.9-37.3)	.57	36.2 (32.0-40.5)	40.5 (39.7-41.4)	.05
Frozen oocytes	20.2 (12.4-28.1)	31.5 (29.3-33.7)	.02	25.0 (12.8-37.3)	34.0 (31.5-36.5)	.13
Thawed embryos	31.9 (27.4-36.5)	3.1 (29.2-31.0)	.40	32.8 (27.6-37.9)	29.8 (28.9-30.8)	.24
Total	33.0 (30.5-35.6)	34.0 (33.5-34.5)	.48	34.2 (31.1-37.2)	36.1 (35.5-36.7)	.23

Abbreviations: HFEA, Human Fertilization and Embryology Authority; SART, Society for Assisted Reproductive Technology.

A binomial logistic regression was performed to explore the association between variables showing significant differences in embryo transfer cycles of women with and without endometriosis (patient’s age, egg donor age, and double donation) and the likelihood of live birth. All of the variables significantly added to the model: embryo transfers in patients aged 45 to 50 years resulted in significantly reduced LBR, whereas embryo transfers involving younger egg donors and double donation recipients had improved success rates. There were no significant differences in LBR and IR according to diagnosis of endometriosis. For LBR per embryo transfer cycle, the crude OR was 0.92 (95% CI, 0.80-1.06), and the adjusted OR was 0.91 (95% CI, 0.79-1.04) for recipients with a diagnosis of endometriosis compared with controls (Figure 1). For IR, the crude OR was 0.96 (95% CI, 0.85-1.08), and the adjusted OR was 0.95 (95% CI, 0.85-1.07) (Figure 2). Detailed results of the logistic regression analysis are reported in Table 3.

**Table 3.** Logistic Regression Estimating Likelihood of Live Birth Based on Independent Variables

Variable^a^	B (SE)	Wald	*df*	*P* value	OR (95% CI)
Patient age, y	NA	34.24	5	.01	NA
35-37	0.01 (0.05)	0.06	1	.81	1.01 (0.92-1.12)
38-39	0.04 (0.05)	0.71	1	.40	1.05 (0.94-1.16)
40-42	0.05 (0.04)	1.21	1	.27	1.05 (0.96-1.14)
43-44	0.07 (0.05)	2.53	1	.11	1.08 (0.98-1.18)
45-50	−0.14 (0.04)	10.72	1	.001	0.87 (0.80-0.95)
Egg donor age, y	NA	39.83	4	.001	NA
21-25	0.18 (0.09)	4.02	1	.05	1.20 (1.00-1.44)
26-30	0.22 (0.09)	5.86	1	.02	1.24 (1.04-1.48)
31-35	0.07 (0.09)	0.63	1	.43	1.07 (0.90-1.28)
>35	−0.15 (0.12)	1.66	1	.20	0.86 (0.68-1.08)
Double donation	0.10 (0.03)	7.79	1	.005	1.10 (1.03-1.18)

Abbreviations: NA, not applicable; OR, odds ratio.

^a^ Reference values include patient age 18 to 34 years, egg donor age 20 years or younger, and data from the Human Fertilization and Embryology Authority register.

#### Pooled Analysis of Data From Registries

Data retrieved from registries were pooled for the outcomes LBR and IR. The unadjusted model showed a significant reduction in LBR per embryo transfer in women with a history of endometriosis (OR, 0.89; 95% CI, 0.82 to 0.97), with an RD of −0.03 (95% CI, −0.05 to −0.01) (Figure 1A). Similar findings were observed after incorporating adjusted data from HFEA into the analysis (OR, 0.89; 95% CI, 0.81 to 0.97) (Figure 1B). With regard to the IR, we observed no significant reduction in women with endometriosis, both in the analysis of raw data (OR, 0.90; 95% CI, 0.80 to 1.01; RD, −0.02; 95% CI, −0.05 to 0.00) (Figure 2A) and after the inclusion of adjusted data from the HFEA registry (OR, 0.90; 95% CI, 0.80 to 1.00) (Figure 2B).

## Discussion

To our knowledge, this is the first systematic review and meta-analysis aimed at determining whether endometriosis is associated with the success rates of donor ART cycles. To comprehensively examine this topic, we conducted a systematic literature review and searched available IVF registries.^19^ Our study included data from 4 published studies and 2 IVF registries, encompassing 7212 patients and 162 082 cycles (24 900 from HFEA and 137 182 from SART). No significant difference in LBR in patients with a history of endometriosis compared with unaffected women was found in the analysis of raw data, with absolute percentage differences of −1.9% and −3.4%, respectively, from the HFEA data set and SART registry and an RD of −0.06 (95% CI −0.15 to 0.03) from the meta-analysis of published studies. However, the evidence quality from the literature analysis was classified as low because of a high risk of imprecision bias. Registries, on the other hand, usually provide more generalizable data but have limits in terms of the variables collected and interpretation. Thus, in estimating the pooled data, considering the diverse data sources and in order to avoid unintentional bias, we have decided to separately aggregate literature and registry data.^20^ Intriguingly, a substantial consistency emerged among the results of the various analyses, although statistical significance was achieved only in the individual analysis of SART (OR, 0.87; 95% CI, 0.78 to 0.98) and in the pooled analysis of registry data, with an unadjusted OR of 0.89 (95% CI, 0.82 to 0.97) and an OR of 0.89 (95% CI, 0.81 to 0.97) after the inclusion of adjusted data. An estimated RD of −0.03 (95% CI, −0.05 to −0.01) deriving from aggregate data from registries supports the concordance in the estimates between the different types of sources.

Some aspects need to be considered when interpreting our findings from a statistical perspective. The sample size from each source represents a crucial factor. It is noteworthy that, despite results similar to those from other sources, the SART database alone, with a sample size 5.5 times larger than HFEA registry and approximately 19 times larger than that of published data, could demonstrate a statistically significant result. No measurable impact on aggregate results was observed when introducing adjusted data into the analyses, confirming the findings of the unadjusted model and highlighting the important influence of the sample size on our statistical comparison. Although a large sample size provides the advantage of increased statistical power, enhancing the ability to detect real effects or differences between groups, it may have the drawback of contributing information of poor clinical significance or practical relevance owing to their small effect sizes. The present results indeed found a small decrease in LBR in women with a history of endometriosis compared with those without. In our case, the modest magnitude of the reduction may be reassuring regarding the effectiveness of donor ART cycles in this population, as severe defects of uterine receptivity in endometriosis are not supported by the present findings.

The biological plausibility underlying impaired uterine receptivity in women with endometriosis is substantial. The eutopic endometrium of women with endometriosis exhibits several molecular and functional abnormalities compared with that of healthy women.^9^ Therefore, findings from basic studies support the notion that endometriosis leads to implantation defects and reduced LBR.

On the other hand, results from basic studies lack clinical confirmation. Considering that endometriosis is a common condition (estimated to affect 10% of reproductive-aged women^21^) and that 37% of patients with endometriosis experience infertility,^22^ one would expect a substantial volume of data to address our study question. Conversely, studies on this topic are limited, dated, and characterized by small sample sizes. Even data from registries have issues related to sample size. When examining the proportions of women with endometriosis and controls in the registries we analyzed (with a ratio of approximately 1:118 in SART and 1:26 in HFEA), it appears that women with endometriosis undergo donor cycles less frequently compared with women with other infertility indications. This seems unlikely, given that autologous ART treatments are needed in approximately 10% to 25% of women with endometriosis,^27^ and endometriosis accounts for approximately 15% of all indications for autologous IVF procedures in ART centers.^28^

The most plausible reason for the imbalance between the anticipated and documented cases of endometriosis in clinical donor registries is that the diagnosis of endometriosis might be overshadowed by other conditions that frequently coincide with the disease, such as poor ovarian response, premature ovarian insufficiency, and early menopause. In other words, the diagnosis of endometriosis might be prioritized less frequently in favor of indications more closely linked to ovarian function at the time of consultation for the donor cycle, because these indications represent the primary biological reason for considering the use of donor oocytes over autologous ones. If this hypothesis holds true, it is possible that, in the registry data sets, many women with endometriosis may be concealed within the control group. Thus, caution is advised in interpreting our pooled estimates.

As far as we know, our study incorporates the first meta-analysis on this topic and is the most comprehensive attempt to synthesize evidence regarding the impact of a history of endometriosis on the outcomes of oocyte donation cycles. The main strengths of our study are represented by its originality and an inclusive and comprehensive design that, with analysis of data from SART and HFEA registries, provides a realistic interpretation gathering the maximum amount of evidence available from the general population.

### Limitations

Our study has limitations inherent to the data analyzed. Regarding the meta-analysis, these limitations include a low number of studies, most of which are old, with a small number of analyzed patients, and the prevalent transfer of multiple embryos at the cleavage stage. For registry data, the main issues are related to the nature of registries themselves, which may have biases related to data compilation, data incompleteness, and the lack of specific patients’ information. It is essential to consider these aspects when interpreting our results. Furthermore, we acknowledge the limitation of our data set in lacking detailed information on participants’ menopausal status, which represents a potential confounder. The menopausal status may attenuate the detrimental effects of endometriosis but, most importantly, it could introduce a bias due to a possible higher proportion of menopausal women in the control group. A consequent lower endometrial receptivity could characterize this group.^29^ This aspect may be crucial in interpreting the variations in reproductive outcomes among different age groups in our study, underscoring the need to consider menopausal status in future research. Along the same line, we have to underline that the presence of some uterine pathologies may concur to partially explain our findings in relation to age. It is well known that the incidence of fibroids increases with age, and the common perception for adenomyosis is that it affects older reproductive-age women.^3,30^ By modifying the vascular architecture, impairing the normal contractility, and changing the production of angiogenic factors, adenomyosis and uterine fibroids might alter local and distant endometrial milieu and, consequently, endometrial function.^3,31^ Unfortunately, we did not have information on this clinical aspect. Notably, the presence of adenomyosis also represents an important confounder in the interpretation of our results that supports an impact of endometriosis on ART success rates in oocyte donor cycles.^3^ Because endometriosis is commonly associated with adenomyosis, failing to highlight a marked detrimental effect of endometriosis indirectly supports the view that also adenomyosis does not markedly affect the success rates of these treatments.

## Conclusions

Our findings suggest a potential minimal decrease in the LBR among recipients with a history of endometriosis undergoing oocyte donation ART cycles. Because this approach can provide insights into the impact of the condition on the process of embryo implantation, we can deduce that a marginal impairment of uterine receptivity contributes to infertility mechanisms in women affected by endometriosis. Whether this defect is associated with comorbidities or confounders have to be clarified in future studies.

## Notes

Supplement 1. **eAppendix.** Supplemental Methods **eTable 1.** Modified Newcastle-Ottawa Scoring Items **eTable 2.** Results From Assessment of the Risk of Study Bias **eTable 3.** Results From Assessment of the Quality of Evidence **eFigure 1.** PRISMA Flow Diagram **eFigure 2.** Forest Plots for Live Birth Rate for the Unadjusted (A) and Mixed Models (B) Subgroup Analysis Based on the Type of Oocyte Donation Cycles (Fresh vs. Frozen) **eTable 4.** Comparison of Main Characteristics of Women With and Without Endometriosis Included in the HFEA Register **eTable 5.** Live Birth Rates and Implantation Rates in Women With and Without Endometriosis Undergoing Donor Egg Cycles Across Various Age Categories Based on the HFEA Dataset

Supplement 2.
Data Sharing Statement
