# Supplementary material for: Live Birth After Oocyte Donation In Vitro Fertilization Cycles in Women With Endometriosis: A Systematic Review and Meta-Analysis
Source: JAMA Netw Open. 2024 Jan 31;7(1):e2354249. doi: 10.1001/jamanetworkopen.2023.54249 (PMC10831577; doi:10.1001/jamanetworkopen.2023.54249)
Supplement: Supplement 1. — eAppendix. Supplemental Methods eTable 1. Modified Newcastle-Ottawa Scoring Items eTable 2. Results From Assessment of the Risk of Study Bias eTable 3. Results From Assessment of the Quality of Evidence eFigure 1. PRISMA Flow Diagram eFigure 2. Forest Plots for Live Birth Rate for the Unadjusted (A) and Mixed Models (B) Subgroup Analysis Based on the Type of Oocyte Donation Cycles (Fresh vs. Frozen) eTable 4. Comparison of Main Characteristics of Women With and Without Endometriosis Included in the HFEA Register eTable 5. Live Birth Rates and Implantation Rates in Women With and Without Endometriosis Undergoing Donor Egg Cycles Across Various Age Categories Based on the HFEA Dataset [file jamanetwopen-e2354249-s001.pdf]

Supplemental Online Content

Paffoni A, Casalechi M, De Ziegler D, et al. Live birth after oocyte donation in vitro fertilization cycles in women with endometriosis: a systematic review and meta-analysis. *JAMA Netw Open*. 2024;7(2):e2354249. doi:10.1001/jamanetworkopen.2023.54249

eAppendix. Supplemental Methods

eTable 1. Modified Newcastle-Ottawa Scoring Items

eTable 2. Results From Assessment of the Risk of Study Bias

eTable 3. Results From Assessment of the Quality of Evidence

eFigure 1. PRISMA Flow Diagram

eFigure 2. Forest Plots for Live Birth Rate for the Unadjusted (A) and Mixed Models (B) Subgroup Analysis Based on the Type of Oocyte Donation Cycles (Fresh vs. Frozen)

eTable 4. Comparison of Main Characteristics of Women With and Without Endometriosis Included in the HFEA Register

eTable 5. Live Birth Rates and Implantation Rates in Women With and Without Endometriosis Undergoing Donor Egg Cycles Across Various Age Categories Based on the HFEA Dataset

This supplemental material has been provided by the authors to give readers additional information about their work.

eAppendix. Supplemental Methods

Information Sources, Search, and Eligibility Criteria

We conducted a comprehensive search of electronic databases, including Sciencedirect, Pubmed, Scopus, Embase, the Cochrane library, Clinicaltrials.gov, EU Clinical Trials Register, and World Health Organization International Clinical Trials Registry, until August 31, 2023, without date restrictions. Two authors (A.V., M.C.) independently assessed the electronic search and study eligibility. Our search terms included endometriosis, endometrioma, donor IVF, oocyte donation, oocyte recipient, and heterologous IVF. After removing duplicates, we screened citations based on titles and abstracts. Additionally, we manually searched the references of included studies to ensure we did not miss any relevant research. Any disagreements were resolved through consensus, and selected items were thoroughly evaluated for eligibility in full-text.

Study selection and Data Collection Process

The inclusion of studies was based on the following criteria: oocyte donation cycles with donors under 35 years of age unaffected by endometriosis; comparison between women with endometriosis versus without endometriosis; reported frequencies of women achieving clinical pregnancy rate or live birth in the two groups; studies reported in the English language; full-text studies. The following exclusion criteria were applied: case reports, case series, non-controlled studies; non-original or duplicated data.

The data extraction process was conducted by two authors (A.V., M.C.) who collected information on study characteristics, populations (number of patients and inclusion criteria), embryo transfer cycle (protocol for endometrial preparation, luteal phase support), and study outcomes. Outcome data were obtained from the text and/or tables of the original studies. If outcome data were not explicitly mentioned, they were calculated based on other available outcome data (e.g., miscarriage rate calculated as the difference between clinical and ongoing pregnancies). In case of missing data, the authors of the original studies were contacted to obtain additional information relevant to the outcomes of our study. One author (P.V.) reviewed the entire data extraction process.

Assessment of the risk of bias

Two reviewers (A.V., M.C.) independently judged the methodological quality of studies included in the meta-analysis using a modified version of the "Newcastle-Ottawa Scale". Quality of the studies was evaluated in five different domains: "*sample representativeness*", "*sampling technique*", "*ascertainment of endometriosis diagnosis*", "*quality of description of the population*", "*incomplete outcome data*"**.** According to the total number of points assigned, each study was judged to be at low risk of bias (≥3 points) or high risk of bias (<3 points). Any discrepancies concerning Author's judgements were referred to a third reviewer (P.V.) and resolved by consensus.

© 2024 Paffoni A et al. *JAMA Network Open.*

Grading of evidence

The body of evidence was assessed by two Authors (A.V., A.P.) using the GRADE (Grading of Recommendations Assessment Development and Evaluation working group) methodology. The final score was obtained by evaluating the following domains: *study design*, *risk of bias*, *indirectness*, *inconsistency*, *imprecision*, *large effect size*, *plausible confounding* and *publication bias*. *Dose response gradient* was not evaluated since the intervention was dichotomous.

© 2024 Paffoni A et al. *JAMA Network Open.*

eTable 1: Modified Newcastle-Ottawa Scoring Items.

(1) Sample representativeness:
1 point: Sample size was greater than or equal to 100 participants per group.
0 points: Sample size was less than 100 participants per group.
(2) Sampling technique:
1 point: Patients recruited consecutively or randomly, with or without matching techniques.
0 points: Potential convenience sampling or unspecified sampling technique.
(3) Ascertainment of endometriosis diagnosis:
1 point: The criteria for the diagnosis of endometriosis were clearly described and included the use of one or more of the following techniques, including transvaginal ultrasound, magnetic resonance imaging, and laparoscopy.
0 points: The diagnosis was based on other unvalidated approaches (e.g., chronic pelvic pain), or the diagnostic methods were not described.
(4) Quality of population description:
1 point: The study reported a clear description of the population (e.g. age, BMI, duration of infertility) with proper measures of dispersion (e.g., mean, standard deviation) and ovarian stimulation-embryo transfer protocol.
0 points: The study did not report a clear description of the population or ovarian stimulation-embryo transfer protocol, incompletely reported descriptive statistics, or did not report measures of dispersion.
(5) Incomplete outcome data:
1 point: The study reported complete data about the primary outcome of the review (live birth rate).
0 points: Selective data reporting could not be excluded.

Legend: The individual components listed above are summed to generate a total modified Newcastle-Ottawa risk of bias score for each study. Total scores range from 0 to 5.
For the total score grouping, studies were judged to be of low risk of bias (≥3 points) or high risk of bias (<3 points).

© 2024 Paffoni A et al. *JAMA Network Open.*

eTable 2: Results From Assessment of the Risk of Study Bias

STUDY ID	SAMPLE REPRESENTA-TIVENESS	SAMPLING TECHNIQUE	ASCERTAIN-MENT OF ENDOMETRIOSIS DIAGNOSIS	QUALITY OF DESCRIPTION OF THE POPULATION	INCOMPLETE OUTCOME DATA	TOTAL SCORE	RISK OF BIAS
Diaz et al., 2010	-	-	★	★	★	★★★	LOW
Kamath et al., 2022	★	★	-	-	★	★★★	LOW
Prapas et al., 2012	★	-	★	★	★	★★★★	LOW
Sung et al., 1997	-	★	★	-	-	★★	HIGH

© 2024 Paffoni A et al. *JAMA Network Open.*

eTable 3: Results From Assessment of the Quality of Evidence

Endometriosis compared to Controls in oocyte donation cycles

Patient or population: oocyte donation cycles

Intervention: Endometriosis

Comparison: Controls

Outcomes	Anticipated absolute effects* (95% CI)		Relative effect (95% CI)	№ of participants (studies)	Certainty of the evidence (GRADE)	Comments
	**Risk with Controls**	**Risk with Endometriosis**				
Live birth rate (LBR)	311 per 1.000	**255 per 1.000** (190 to 333)	**OR 0.76** (0.52 to 1.11)	6973 (3 observational studies)	⨁⨁◯◯ Low[a]	
Clinical Pregnancy Rate (CPR)	409 per 1.000	**327 per 1.000** (250 to 417)	**OR 0.70** (0.48 to 1.03)	537 (3 observational studies)	⨁◯◯◯ Very low[b,c]	
Implantation Rate (IR)	189 per 1.000	**152 per 1.000** (121 to 189)	**OR 0.77** (0.59 to 1.00)	1679 (3 observational studies)	⨁◯◯◯ Very low[b,c]	
Miscarriage Rate (MR)	153 per 1.000	**191 per 1.000** (90 to 361)	**OR 1.31** (0.55 to 3.13)	149 (2 observational studies)	⨁◯◯◯ Very low[d]	

***The risk in the intervention group** (and its 95% confidence interval) is based on the assumed risk in the comparison group and the **relative effect** of the intervention (and its 95% CI).

CI: confidence interval; **OR:** odds ratio

GRADE Working Group grades of evidence
High certainty: we are very confident that the true effect lies close to that of the estimate of the effect.
Moderate certainty: we are moderately confident in the effect estimate: the true effect is likely to be close to the estimate of the effect, but there is a possibility that it is substantially different.
Low certainty: our confidence in the effect estimate is limited: the true effect may be substantially different from the estimate of the effect.
Very low certainty: we have very little confidence in the effect estimate: the true effect is likely to be substantially different from the estimate of effect.

Explanations

a. Higgins I^2 value between 30% and 60% indicating moderate statistical heterogeneity in the study results.

b. Low number of patients and events. Two studies included in the analysis utilized an older clinical and laboratory standard (studies published in 1997 and 2000).

c. All the studies performed multiple embryo transfers at the cleavage stage (ranging from 2 to 4 embryos per single transfer).

d. Low number of events and pregnancies. High risk of twin pregnancies due to multiple embryo transfers. Significant age differences between patients with endometriosis and those without endometriosis (women with endometriosis older on average).

© 2024 Paffoni A et al. *JAMA Network Open.*

eFig.1 PRISMA Flow-Diagram

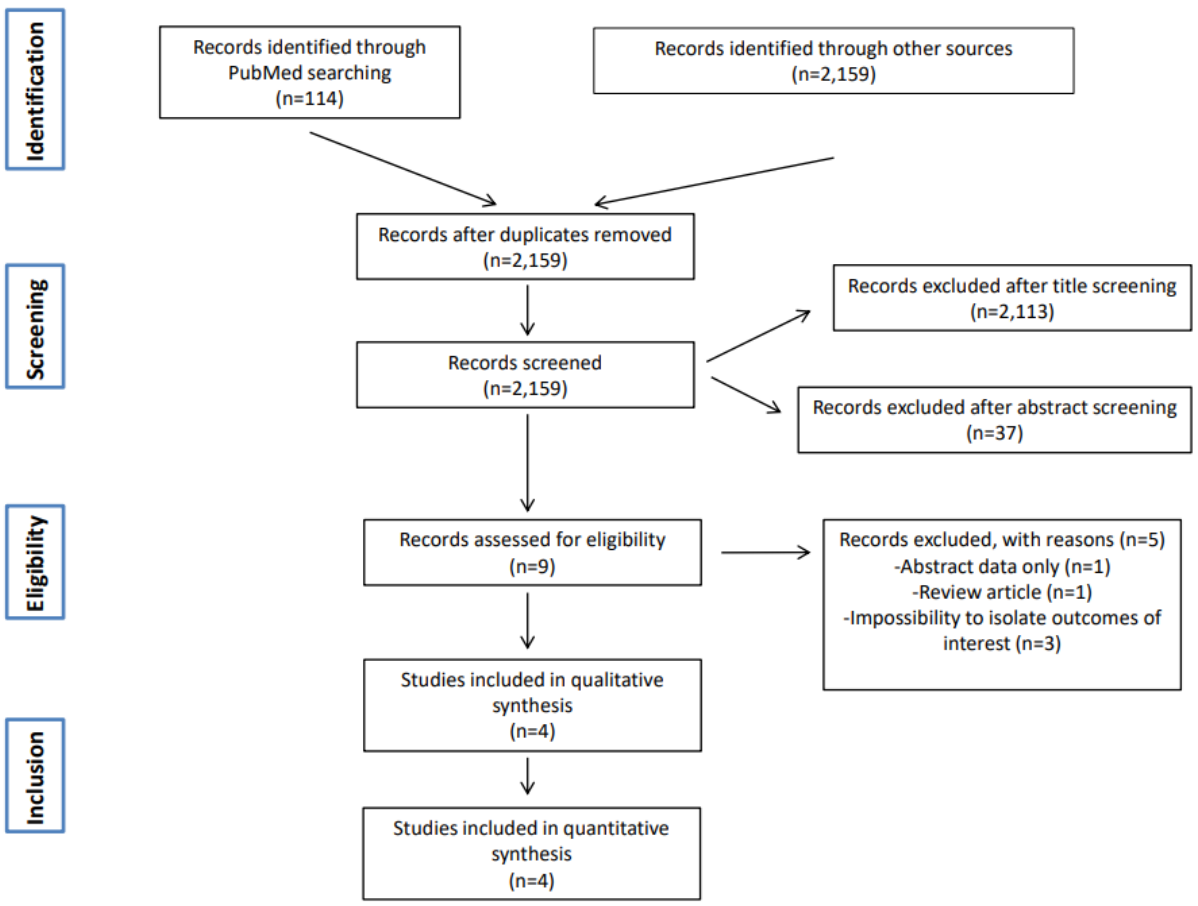

eFigure 1. PRISMA Flow Diagram

© 2024 Paffoni A et al. *JAMA Network Open.*

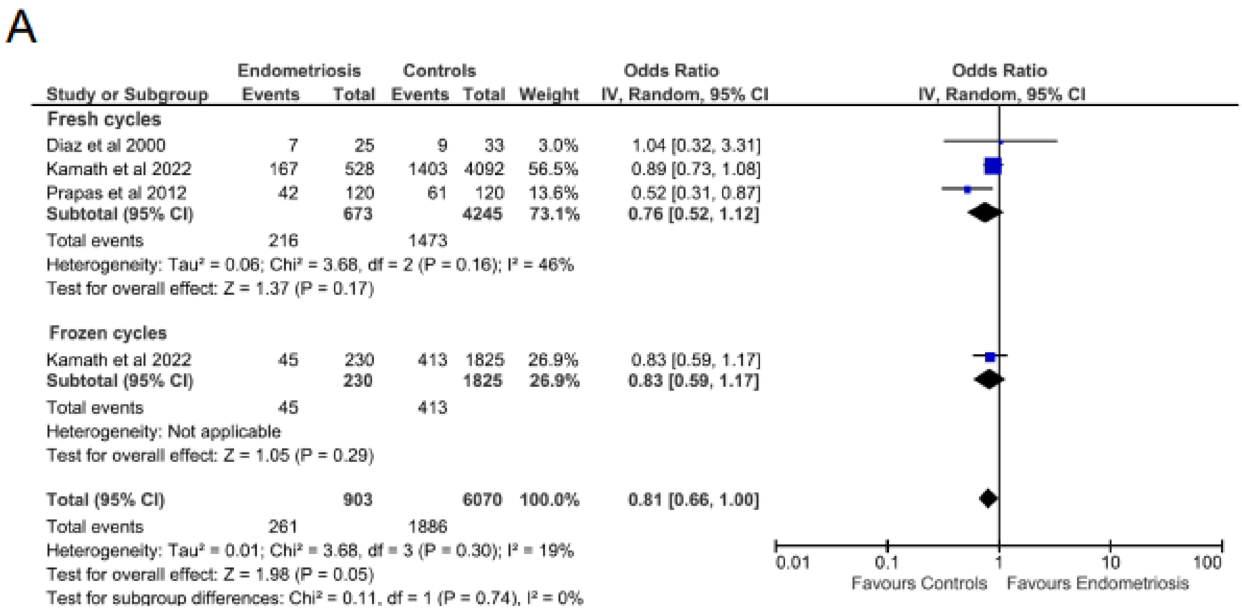

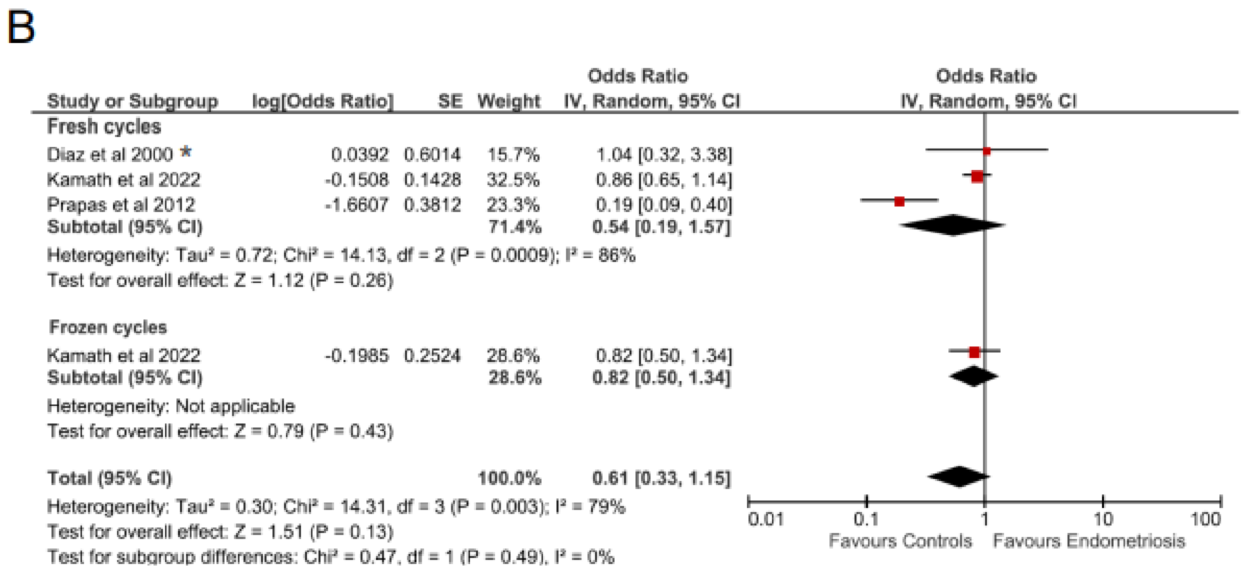

eFigure 2. Forest plots for live birth rate for the unadjusted (A) and mixed models (B) subgroup analysis based on the type of oocyte donation cycles (fresh vs. frozen). The asterisk refers to non-adjusted data. Blue squares refer to dichotomous variables presented in A. Red squares indicate the inclusion of log odds ratio and the standard error of the log odds ratio in the meta-analysis shown in B. Size of the squares depends on the weight assigned to each study in the analysis.

© 2024 Paffoni A et al. *JAMA Network Open.*

eTable 4: Comparison of Main Characteristics of Women With and Without Endometriosis Included in the HFEA Register.

	Endometriosis, Total=931		Controls, Total=23969		*p*
	n	%	n	%	
Patient age					0.001
Patient age (18-34 years)	145	15.6%	4419	18.4%	
Patient age (35-37 years)	157	16.9%	2685	11.2%	
Patient age (38-39 years)	118	12.7%	2328	9.7%	
Patient age (40-42 years)	248	26.6%	4709	19.6%	
Patient age (43-44 years)	131	14.1%	3779	15.8%	
Patient age (45-50 years)	132	14.2%	6049	25.2%	
Egg donor age					0.001
Egg donor age (<21 years)	17	1.8%	597	2.5%	
Egg donor age (21-25 years)	194	20.8%	4058	16.9%	
Egg donor age (26-30 years)	333	35.8%	8119	33.9%	
Egg donor age (31-35 years)	376	40.4%	10481	43.7%	
Egg donor age (>35 years)	11	1.2%	714	3.0%	
Patients with previous ART live births	123/893*	13.8%	3310/22292*	14.8%	0.38
Presence of a male factor	204	21.9%	4875	20.3%	0.24
N° embryos transferred					0.74
1	556	59.7%	14344	59.8%	
2	375	40.3%	9610	40.1%	
3	0	0.0%	15	0.1%	
Year of treatment					0.93
2010-2014	425	45.6%	10979	45.8%	
2015-2018	506	54.4%	12990	54.2%	
Type of cycle					0.96
Fresh oocytes	516	55.4%	13337	55.6%	
Frozen oocytes	64	6.9%	1588	6.6%	
Thawed embryos	351	6.9%	9044	37.7%	
Double donation	49	5.3%	5018	20.9%	0.001

* Missing information in 38 cases and 1677 controls. ART: Assisted Reproductive Technologies

© 2024 Paffoni A et al. *JAMA Network Open.*

eTable 5. Live birth rates and implantation rates in women with and without endometriosis undergoing donor egg cycles across various age categories based on the HFEA dataset

			2010-2014		2015-2018		Total	
Patients' age, n	Outcome		Endometriosis	Controls	Endometriosis	Controls	Endometriosis	Controls
18-34 years,	Live birth	n	18	667	28	950	46	1617
n=4564	occurrence	%	23,7%	35,0%	40,6%	37,8%	31,7%	36,6%
	Number of	0	50	1161	23	771	73	1932
	foetal sacs	1	21	601	30	969	51	1570
	with pulsation	2	5	145	5	123	10	268
		3	0	1	0	1	0	2
	Embryos	1	36	979	44	1889	80	2868
	transferred	2	40	926	25	622	65	1548
		3	0	3	0	0	0	3
	Crude Implantation		26,7%	31,5%	42,6%	38,9%	33,8%	35,4%
35-37 years,	Live birth	n	27	437	27	536	54	973
n=2842	occurrence	%	38,6%	35,6%	31,0%	36,7%	34,4%	36,2%
	Number of	0	41	747	34	468	75	1215
	foetal sacs	1	22	386	28	573	50	959
	with pulsation	2	7	93	3	47	10	140
		3	0	0	0	2	0	2
	Embryos	1	33	566	54	1083	87	1649
	transferred	2	37	660	33	375	70	1035
		3	0	0	0	1	0	1
	Crude Implantation		33,6%	30,3%	28,3%	36,7%	30,8%	33,4%
38-39 years,	Live birth	n	17	406	25	456	42	862
n=2446	occurrence	%	32,1%	36,2%	38,5%	37,8%	35,6%	37,0%
	Number of	0	34	669	24	384	58	1053
	foetal sacs	1	16	349	29	476	45	825
	with pulsation	2	3	102	3	44	6	146
		3	0	2	0	1	0	3
	Embryos	1	22	459	45	865	67	1324
	transferred	2	31	662	20	341	51	1003
		3	0	1	0	0	0	1
	Crude Implantation		26,2%	31,3%	41,2%	36,7%	33,7%	33,8%
40-42 years,	Live birth	n	39	831	44	928	83	1759
n=4957	occurrence	%	33,3%	36,3%	33,6%	38,3%	33,5%	37,4%
	Number of	0	74	1345	39	831	113	2176
	foetal sacs	1	37	748	51	951	88	1699
	with pulsation	2	6	193	3	118	9	311
		3	0	1	0	3	0	4
	Embryos	1	57	931	106	1709	163	2640
	transferred	2	60	1354	25	711	85	2065
		3	0	3	0	1	0	4
	Crude Implantation		27,7%	31,2%	36,5%	38,2%	31,8%	34,4%
43-44 years,	Live birth	n	17	652	32	781	49	1433
n=3910	occurrence	%	37,8%	37,6%	37,2%	38,2%	37,4%	37,9%
	Number of	0	28	998	37	712	65	1710
	foetal sacs	1	13	576	28	793	41	1369
	with pulsation	2	4	158	7	95	11	253
		3	0	3	0	1	0	4
	Embryos	1	23	729	58	1477	81	2206
	transferred	2	22	1006	28	566	50	1572
		3	0	0	0	1	0	1
	Crude Implantation		31,3%	32,9%	36,8%	37,7%	34,8%	35,3%
45-50 years,	Live birth	n	22	882	22	1120	44	2002
n=6181	occurrence	%	34,4%	32,7%	32,4%	33,4%	33,3%	33,1%
	Number of	0	39	1680	20	1239	59	2919
	foetal sacs	1	16	828	28	1172	44	2000
	with pulsation	2	9	189	1	156	10	345
		3	0	3	0	2	0	5
		>3	0	0	0	1	0	1
	Embryos	1	29	1218	49	2439	78	3657
	transferred	2	35	1479	19	908	54	2387
		3	0	3	0	2	0	5
	Crude Implantation		34,3%	29,0%	34,5%	35,0%	34,4%	32,1%
Overall	Live birth	n	140	3875	178	4771	318	8646
	occurrence	%	32,9%	35,3%	35,2%	36,7%	34,2%	36,1%
	Crude Implantation		29,7%	30,9%	36,3%	37,1%	33,0%	34,0%

© 2024 Paffoni A et al. *JAMA Network Open.*
