# Supplementary material for: Live Birth After Oocyte Donation In Vitro Fertilization Cycles in Women With Endometriosis: A Systematic Review and Meta-Analysis
Source: JAMA Netw Open. 2024 Jan 31;7(1):e2354249. doi: 10.1001/jamanetworkopen.2023.54249 (PMC10831577; doi:10.1001/jamanetworkopen.2023.54249)
Supplement: Supplement 2. — Data Sharing Statement [file jamanetwopen-e2354249-s002.pdf]

Data Sharing Statement

Paffoni. Live Birth After Oocyte Donation In Vitro Fertilization Cycles in Women With Endometriosis. *JAMA Netw Open*. Published February 01, 2024.
doi:10.1001/jamanetworkopen.2023.54249

Data
Data available: Yes
Data types: Deidentified participant data
How to access data: https://www.sart.org/ https://www.hfea.gov.uk/
When available: With publication

Supporting Documents
Document types: None

Additional Information
Who can access the data: data are publicly available. Template data collection forms and data extracted from studies can be requested to the corresponding Author.
Types of analyses: for research purposes.
Mechanisms of data availability: after reasonable request
Any additional restrictions: data are publicly available
